# Comparison of Two Circular-Stapled Techniques for Esophageal Cancer: A Propensity-Matched Analysis

**DOI:** 10.3389/fonc.2021.759599

**Published:** 2021-12-16

**Authors:** Hang Lin, Ge’ao Liang, Huiping Chai, Yongde Liao, Chunfang Zhang, Yuanda Cheng

**Affiliations:** ^1^ Department of Thoracic Surgery, Xiangya Hospital, Central South University, Changsha, China; ^2^ Department of Oncology, National Health Commission (NHC) Key Laboratory of Cancer Proteomics, Xiangya Hospital, Central South University, Changsha, China; ^3^ Department of Burns and Plastic Surgery, Third Xiangya Hospital, Central South University, Changsha, China; ^4^ Department of Thoracic Surgery, First Affiliated Hospital of Anhui Medical University, Hefei, China; ^5^ Department of Thoracic Surgery, Tongji Hospital, Tongji Medical College, Huazhong University of Science and Technology, Wuhan, China; ^6^ Human Engineering Research Center for Pulmonary Nodules Precise Diagnosis and Treatment, Xiangya Hospital, Central South University, Changsha, China; ^7^ National Clinical Research Center for Geriatric Disorders, Xiangya Hospital, Central South University, Changsha, China

**Keywords:** esophageal cancer, minimally invasive Ivor Lewis esophagectomy, thoracoscopic intrathoracic esophagogastric anastomosis, anastomotic technique, anastomotic leakage

## Abstract

**Objective:**

The optimal technique for the thoracoscopic construction of an intrathoracic esophagogastric anastomosis continues to be a subject of controversy. The aim of this study was to compare the perioperative outcomes of circular-stapled anastomosis using a transorally inserted anvil (Orvil™) with those of circular-stapled anastomosis using a transthoracically placed anvil (non-Orvil™) in totally minimally invasive Ivor Lewis esophagectomy (Ivor Lewis TMIE).

**Methods:**

The data of 272 patients who underwent Ivor Lewis TMIE for esophageal cancer at multiple centers were collected from January 1, 2014 to December 31, 2017. After propensity score matching (1:1) for patient baseline characteristics, 65 paired cases were selected for statistical analysis. Logistic regression analysis was performed to investigate the significant factors of anastomotic leakage.

**Results:**

In the propensity score-matched analysis, compared with the non-Orvil™ group, the Orvil™ group was associated with a significantly shorter operation time (*p*=0.031), less intraoperative hemorrhage (*p*<0.001), lower need for intraoperative transfusions (*p*=0.009), earlier postoperative oral feeding time (*p*=0.010), longer chest tube duration (*p*<0.001), shorter postoperative hospital stays (*p*=0.001), lower total hospitalization costs (*p*<0.001) and a lower postoperative anastomotic leakage rate (*p*=0.033). Multivariate logistic regression analysis showed that anastomotic technique and pulmonary infection were independent factors for the development of postoperative anastomotic leakage (*p*< 0.05).

**Conclusions:**

Orvil™ anastomosis exhibited better perioperative effects than non-Orvil™ anastomosis after the propensity score-matched analysis. Remarkably, the Orvil™ technique contributed to a lower postoperative anastomotic leakage rate than the non-Orvil™ technique.

## Introduction

Esophageal cancer is the sixth leading cause of cancer-related mortality among both men and women worldwide, accounting for approximately 5.5% of all cancer deaths worldwide in 2020 (1). Due to advancements in management and treatment, the overall 5-year survival of patients with esophageal cancer has improved in recent decades, but the overall prognosis remains poor (2). Nowadays, esophagectomy continues to play an important role in achieving locoregional control in patients with esophageal cancer, and totally minimally invasive Ivor Lewis esophagectomy (Ivor Lewis TMIE) is the mainstay of curative treatment for middle and lower esophageal cancer, decreasing the rates of major morbidities and mortality (3). Anastomotic leakage is one of the most deleterious complications following minimally invasive esophagectomy (MIE) for cancer. Although the rate of intrathoracic anastomotic leakage is low, anastomotic leakage remains unavoidable and is often associated with a significant risk of perioperative morbidities.

The anastomotic technique has always been considered one of the important factors of postoperative anastomotic leakage (4). Currently, the circular-stapled (CS) technique is considered to be a recommendable approach and has been widely used in intrathoracic esophagogastric anastomosis. However, the placement of the anvil into the esophageal stump is the most challenging step of thoracoscopic esophagogastric anastomosis when a circular stapler is used during Ivor Lewis TMIE. Conventional technique for the transthoracic placement of the anvil (non-Orvil™) is increasingly used for intrathoracic esophagogastric anastomosis. A retrospective study of 215 patients undergoing Ivor Lewis TMIE showed that the CS technique with purse-string suture is feasible and safe to perform, and the rate of postoperative anastomotic leakage was only 2.79% (5). Novel technique for the transoral placement of the anvil (Orvil™) was first used for Ivor Lewis TMIE in 2008 (6). The design of the Orvil™ device is innovative and fundamentally transforms the conventional esophagogastric anastomotic technique; primarily, the thoracoscopic esophageal purse-string suture is replaced with a linear-stapled transected esophageal stump, and the anvil is placed transorally rather than transthoracically. The technique improved the technical feasibility and safety of esophagogastric anastomosis during Ivor Lewis TMIE, with preliminary results showing an anastomotic leakage rate of 2.7% (7). Over the last decade, most of the published studies about the Orvil™ technique in Ivor Lewis TMIE have included small sample sizes. A direct comparison between the Orvil™ CS technique and the non-Orvil™ CS technique for esophageal cancer has never been reported. The optimal technique for the thoracoscopic construction of an intrathoracic esophagogastric anastomosis continues to be a subject of controversy. Our objective was to compare the two different anastomotic techniques in terms of perioperative outcomes in a consecutive series of patients who underwent Ivor Lewis TMIE at three regional public hospitals.

## Patients and Methods

### Data Sources and Study Population

**Figure 1:**
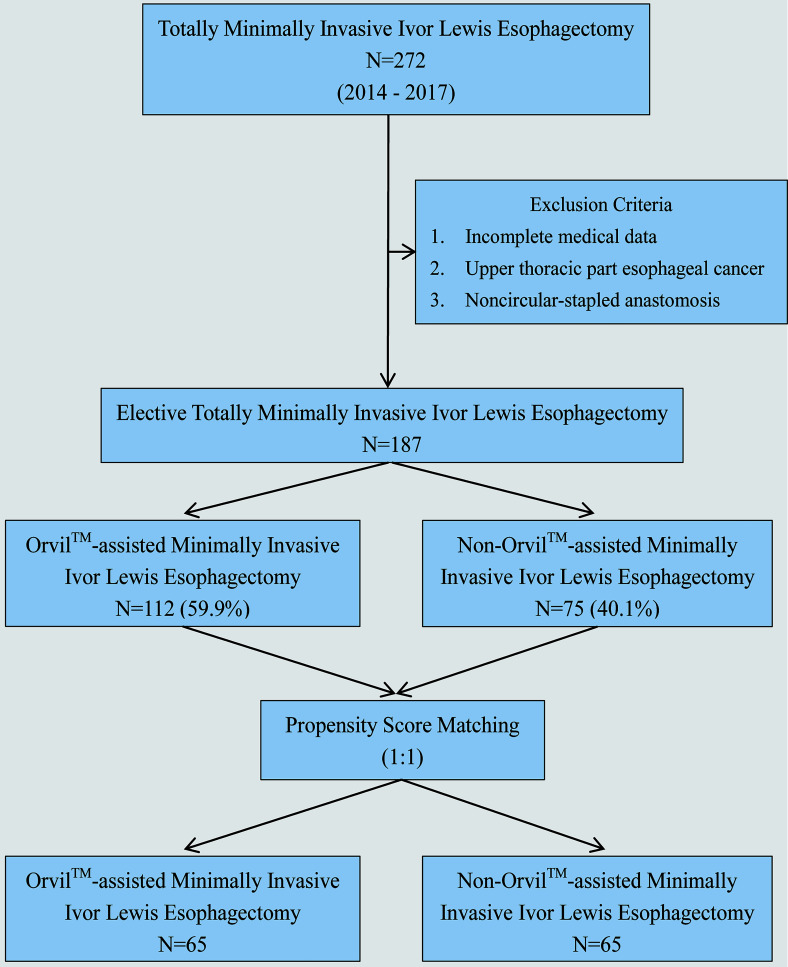
Flowchart for propensity score matching analysis.

We identified 272 consecutive patients undergoing Ivor Lewis TMIE for esophageal cancer from January 1, 2014 through December 31, 2017 at Xiangya Hospital Central South University (X Hospital), Tongji Hospital Affiliated to Tongji Medical College Huazhong University of Science and Technology (T Hospital) and First Affiliated Hospital Anhui Medical University (F Hospital). Patients with noncircular-stapled anastomosis, incomplete medical data, or upper thoracic esophageal cancer were excluded. The remaining 187 patients who underwent Ivor Lewis TMIE constituted the study group, including 112 cases of Orvil™ anastomosis (22 cases from X Hospital and 90 cases from F Hospital) and 75 cases of non-Orvil™ anastomosis (75 cases from T Hospital). To achieve a better homogeneity in subgroups, we excluded several patients by using propensity score matching (**Figure 1**).

The Institutional Review Board of the three hospitals approved this clinical study and granted a waiver of the informed consent process because of the retrospective nature of this study. The data were collected, including patient characteristics (age, sex, body mass index, cigarette smoking, alcohol consumption and comorbidities), tumor characteristics (location, size, type of histology and TNM stage), and perioperative outcomes (preoperative hemoglobin level, preoperative albumin level, operation time, intraoperative hemorrhage, intraoperative transfusion, postoperative chest tube duration, postoperative stomach tube duration, postoperative oral feeding time, length of postoperative hospital stay, postoperative major complications, hospitalization costs). All patients underwent comprehensive preoperative assessments, including laboratory examinations, enhanced computed tomography scans of the chest and abdomen, gastroscopy examinations, positron emission tomography-computed tomography scans, cardiologic examinations and pulmonary functional assessments.

All cases were staged according to the 8^th^ edition of the TNM classification of esophageal cancer (8). Wound infection was defined as superficial or deep surgical site infection in the first seven days after surgery according to the Centers for Disease Control definition (9). Pulmonary infection was defined as new or changed lung opacities and two or more of the following criteria: fever, purulent sputum with cough and/or white blood cell count>12x10^9^/L (9). Anastomotic leakage was defined as any identifiable extravasation at the esophagogastric anastomosis observed by an oral methylene blue test or upper gastrointestinal radiography. Anastomotic stricture was defined as any significant stricture noted by endoscopy or barium contrast medium esophagram during the follow-up, and patients underwent at least one dilation (10). Diagnostic criteria for chylothorax were applied as described by Shah and colleagues (11).

### Anastomotic Procedure

**Figure 2:**
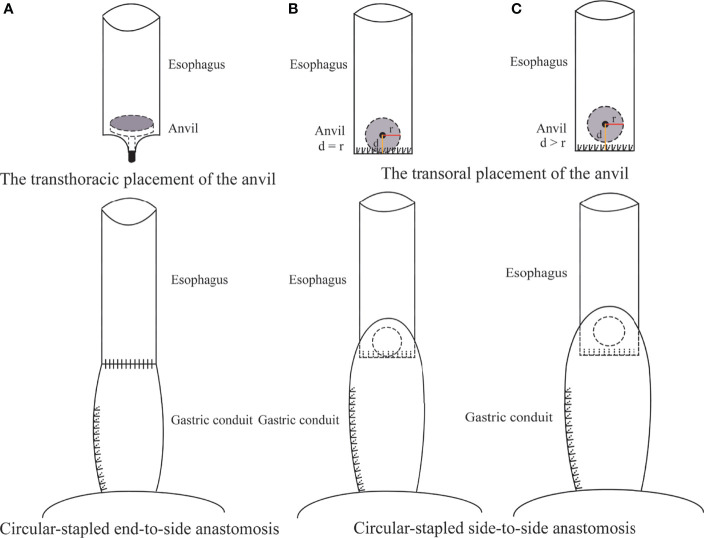
Non-Orvil^TM^ CS anastomosis using a transthoracically placed anvil **(A)** versus Orvil ^TM^ CS anastomosis using a transorally inserted anvil **(B, C)** during Ivor Lewis TMIE. d was defined as the distance between the location of the small opening and the staple line of the esophageal stump. r was defined as the radius of the anvil.

NON-ORVIL™ ANASTOMOSIS. During the thoracic stage, thoracoscopic mobilization of the esophagus to the site located approximately 4 cm above the azygos vein was performed by using a cautery hook and ultrasonic scalpel (12). The thoracoscopic purse-string suture of the esophagus was hand sewn with the 3-0 Surgipro suture that encircled the muscular layer of the esophagus approximately 3 cm above the azygos vein (12). A horizontal incision was made on the wall of the esophagus approximately 3 cm distal to the purse-string to facilitate the passage of the anvil. The 25-mm anvil was inserted into the esophagus through the incision and pushed upward above the purse-string, which was tied by using a knot pusher (12). The distal esophagus and tumor were transected between the purse-string and the incision. The stomach and the gastric conduit were brought into the thoracic cavity. A 25-mm circular stapler (Johnson and Johnson, New Brunswick, New Jersey) was inserted into the side of the gastric conduit, locked into the anvil and tightened (12). The side of the gastric conduit was anastomosed to the end of the esophagus by joining the anvil to the stapler (**Figure 2A**).

ORVIL™ ANASTOMOSIS. During the thoracic phase, the esophagus was mobilized to the area 5 cm superior to the tumor under thoracoscopy, and the proximal esophagus was transected by using a linear stapler (Covidien, New Haven, Connecticut). A 25-mm anvil (Covidien, New Haven, Connecticut) installed on an oral-gastric tube was passed transorally by an experienced assistant. Once the pressure from the oral-gastric tube was observed at the esophageal stump, a small esophagotomy approximately 1 cm perpendicular to the stapled line of the esophageal stump was performed by a hook cautery to facilitate the passage of the oral-gastric tube. Then, the oral-gastric tube was separated from the anvil and removed through a thoracic trocar. The anvil was placed to the side of the esophageal stump in preparation for being connected to the circular stapler. The stomach was pulled upward into the thoracic cavity to remove the esophagus and tumor. A 25-mm circular stapler (Covidien, New Haven, Connecticut) was inserted into the stomach through the cardia and positioned to the side of the stomach. Subsequently, the anvil and circular stapler were connected and then fired to complete the esophagogastric anastomosis (**Figures 2B, C**).

### Statistical Analysis

Propensity score matching was performed to eliminate the differences in patient baseline characteristics to ensure homogeneity and comparability of subgroups. The Orvil™ CS anastomosis and non-Orvil™ CS anastomosis groups were matched one-to-one by a nearest propensity score. The model reliability was appropriate after the efficient matching. The covariates which enrolled in patient characteristics (age, sex, body mass index, cigarette smoking, alcohol consumption and comorbidities) and tumor characteristics (location, size, type of histology and TNM stage) were utilized to estimate the propensity score.

All statistical analyses were performed with SPSS software version 25.0 (IBM, Armonk, NY, USA). The continuous variables that met the criteria of homoscedasticity and normality were expressed as mean ± SD and were further tested using Student’s *t*-test, otherwise, presentation of median (interquartile range) and statistical test of the Mann-Whitney U test were applicable. The categorical variables were expressed as percentages and were compared by either the chi-square test or Fisher’s exact test. To investigate the underlying risk/protective factors of anastomotic leakage, univariate logistic regression analysis was initially performed with the candidate variables, which the significant parameters were further verified by multivariate logistic regression analysis. The statistical significance levels were determined by two-sided tests and a *p* value less than 0.05 was considered to be statistically significant.

## Results

In this study, the patient baseline characteristics before propensity score matching are summarized in **Table 1**. The differences between the two anastomotic techniques underscored the heterogeneity of patient baseline characteristics which mainly showed in age, preoperative albumin level, to tumor location and tumor size with significant differences. Then, of the elective Orvil™ cases (n=112), sixty-five were matched to the non-Orvil™ group. The patient baseline characteristics were similar and not significantly different after propensity score matching (**Table 1**).

**Table 1:** Patient baseline characteristics .

Characteristics	Unmatched patients			Matched patients		
	Orvil™ anastomosis (n=112)	Non-Orvil™ anastomosis (n=75)	*p* Value	Orvil™ anastomosis (n=65)	Non-Orvil™ anastomosis (n=65)	*p* Value
Age, years	64.0 (58.0 - 69.8)	62.0 (54.0 - 66.0)	0.008	62.0 (54.0 - 68.0)	62.0 (54.5 - 67.0)	0.950
Sex, n (%)			0.055			0.784
Male	88 (78.6)	67 (89.3)		58 (89.2)	57 (87.7)	
Body mass index, kg/m^2^	22.0 ± 3.2	23.0 ± 3.3	0.045	22.5 ± 3.0	22.6 ± 2.6	0.854
Cigarette smoking, n (%)			0.083			0.276
Yes	62 (55.4)	51 (68.0)		38 (58.5)	44 (67.7)	
Alcohol consumption, n (%)			0.456			0.725
Yes	55 (49.1)	41 (54.7)		34(52.3)	36 (55.4)	
Comorbidities, n (%)						
Hypertension	24 (21.4)	12 (16.0)	0.356	12 (18.5)	12 (18.5)	1.000
Diabetes	5 (4.5)	1 (1.3)	0.404	1 (1.5)	1 (1.5)	1.000
Cerebrovascular disease	7 (6.3)	7 (9.3)	0.432	6 (9.2)	7 (10.8)	0.770
Peptic ulcer	7 (6.3)	5 (6.7)	1.000	6 (9.2)	5 (7.7)	0.753
Preoperative hemoglobin level, mg/dL	134.0 (121.3 - 142.0)	135.0 (126.0 - 145.0)	0.279	137.0 (122.0 - 147.0)	134.0 (125.0 - 143.5)	0.670
Preoperative albumin level, mg/dL	41.5 ± 5.1	38. 8 ± 5.2	<0.001	40.2 ± 5.0	39.0 ± 5.5	0.190
Tumor location, n (%)			0.036			0.441
Middle thoracic part	45 (40.2)	19 (25.3)		21 (32.3)	17 (26.2)	
Lower thoracic part	67 (59.8)	56 (74.7)		44 (67.7)	48 (73.8)	
Tumor size, cm	3.5 (2.5 - 4.5)	4.0 (3.0 - 5.0)	<0.001	3.7 (2.9 - 5.0)	4.0 (3.0 - 5.0)	0.071
Tumor histology, n (%)			0.927			0.910
Squamous cell carcinomas	101 (90.2)	69 (92.0)		57 (87.7)	59 (90.8)	
Adenocarcinoma	6 (5.4)	3 (4.0)		5 (7.7)	3 (4.6)	
Other	5 (4.5)	3 (4.0)		3 (4.6)	3 (4.6)	
Tumor stage, n (%)			0.526			0.464
0	2 (1.8)	2 (2.7)		2 (3.1)	2 (3.1)	
I	17 (15.2)	12 (16.0)		7 (10.8)	9 (13.8)	
II	61 (54.5)	33 (44.0)		37 (56.9)	27 (41.5)	
III	27 (24.1)	26 (34.7)		15 (23.1)	25 (38.5)	
IV	5 (4.5)	2 (2.7)		4 (6.2)	2 (3.1)	

Values are mean ± SD, median (IQR), or n (%).

The clinical outcomes of the propensity matched Orvil™ versus non-Orvil™ groups are described in **Table 2**. Compared with the patients in the non-Orvil™ group, the patients in the Orvil™ group had a significantly shorter operation time (258.8 minutes versus 287.6 minutes, *p*=0.031), less intraoperative hemorrhage (150.0 ml versus 250.0 ml, *p*<0.001), lower need for intraoperative transfusions (7.7% versus 24.6%, *p*=0.009) and earlier oral feeding time following esophagectomy (8 days versus 9 days, *p*=0.010). The postoperative chest tube duration for the Orvil™ group was 4 days longer than that for the non-Orvil™ group (9 days versus 5 days, *p*<0.001), but the postoperative nasogastric tube duration was not significantly different between the two groups (9 days versus 10 days, *p*=0.405). The postoperative hospital stays and the total hospitalization costs were less for the Orvil™ group than for the non-Orvil™ group (11 days versus 14 days, *p*=0.001, and 76,103.8 RMB versus 98,651.3 RMB, *p*<0.001, respectively). The incidence of postoperative major complications associated with the two groups was no significant difference (18.5% versus 26.2%, *p*=0.292). The results of a detailed analysis of the postoperative major complication rates between the Orvil™ group and the non-Orvil™ group are shown in **Table 3**. The incidence of anastomotic leakage was significantly lower in the Orvil™ group than in the non-Orvil™ group (1.5% versus 12.3%, *p*=0.033). The remaining complications did not differ significantly between the two groups.

**Table 2:** Clinical outcomes, matched analysis (1:1).

Variables	Orvil™ anastomosis (n=65)	Non-Orvil™ anastomosis (n=65)	*p* Value
Operation time, minutes	258.8 ± 75.2	287.6 ± 75.5	0.031
Intraoperative hemorrhage, ml	150.0 (50.0 - 300.0)	250.0 (200.0 - 500.0)	<0.001
Intraoperative transfusions, n (%)	5 (7.7)	16 (24.6)	0.009
Length of chest tube duration, days	9.0 (7.0 - 11.5)	5.0 (4.0 - 6.5)	<0.001
Length of nasogastric tube duration, days	9.0 (7.0 - 13.0)	10.0 (9.0 - 12.0)	0.405
Postoperative oral feeding time, days	8.0 (7.0 - 11.0)	9.0 (8.0 - 11.0)	0.010
Postoperative major complications, n (%)	12 (18.5)	17 (26.2)	0.292
Length of postoperative hospital stay, days	11.0 (9.0 - 16.0)	14.0 (11.5 - 16.0)	0.001
Total hospitalization costs, RMB	76,103.8 (64,518.3 - 105,933.7)	98,651.3 (92,804.4 - 110,773.9)	<0.001

Values are median (IQR), or n (%).

**Table 3:** Postoperative major complications, matched analysis (1:1).

Complications	Orvil™ anastomosis (n = 65)	Non-Orvil™ anastomosis (n = 65)	*p* Value
Wound infection, n (%)	6 (9.2)	2 (3.1)	0.273
Anastomotic stricture, n (%)	1 (1.5)	5 (7.7)	0.208
Anastomotic leakage, n (%)	1 (1.5)	8 (12.3)	0.033
Gastric emptying dysfunction, n (%)	2 (3.1)	0 (0.0)	0.496
Arrhythmia, n (%)	0 (0.0)	2 (3.1)	0.496
Pulmonary infection, n (%)	4 (6.2)	6 (9.2)	0.510
Chylothorax, n (%)	1 (1.5)	1 (1.5)	1.000

Values are n (%).

The results of a univariate analysis of the unadjusted anastomotic leakage and nonanastomotic leakage cohorts are displayed in **Supplemental Table 1**. The total rate of anastomotic leakage was 5.3% (10/187). The patients in the anastomotic leakage cohort had significantly longer postoperative hospital stays (23 days versus 12 days, *p*<0.001) and dramatically higher total hospitalization costs (104788.7 RMB versus 86068.2 RMB, *p*=0.005) than the patients in the nonanastomotic leakage cohort. The anastomotic technique and pulmonary infection also differed significantly between the two cohorts. A total of 187 patients were included in the logistic regression model, and the results of the regression analysis are depicted in **Table 4**. The multivariate analysis indicated that anastomotic technique and pulmonary infection were significant factors associated with anastomotic leakage. The odds ratios for the anastomotic technique and pulmonary infection were 0.114 (95% confidence interval [CI], 0.016-0.781; *p*=0.027) and 65.129 (95% CI, 10.717-395.781; *p*<0.001), respectively.

**Table 4:** Logistic regression analysis of variables comparing the anastomotic leakage and nonanastomotic leakage patients.

Variables	n	Univariate analysis		Multivariate analysis	
		OR (95% CI)	*p* Value	OR (95% CI)	*p* Value
Sex			0.546		
Male	155	1			
Female	32	0.523 (0.064 - 4.282)			
Cigarette smoking			0.084		
No	74	1			
Yes	113	6.317 (0.783 - 50.950)			
Alcohol consumption			0.237		
No	91	1			
Yes	96	2.307 (0.578 - 9.209)			
Hypertension			0.951		
No	151	1			
Yes	36	1.051 (0.214 - 5.177)			
Cerebrovascular disease			0.757		
No	173	1			
Yes	14	1.402 (0.165 - 11.935)			
Peptic ulcer			0.638		
No	175	1			
Yes	12	1.677 (0.194 - 14.456)			
Tumor location			0.773		
Middle thoracic part	64	1			
Lower thoracic part	123	1.227 (0.306 - 4.914)			
Anastomotic techniques			0.019		0.027
Non-Orvil™ anastomosis	75	1		1	
Orvil™ anastomosis	112	0.152 (0.031 - 0.739)		0.114(0.016 - 0.781)	
Pulmonary infection			<0.001		<0.001
No	176	1		1	
Yes	11	51.600 (10.992 - 242.229)		65.129(10.717 - 395.781)	

OR, odds ratio; CI, confidence interval.

## Discussion

In this study, the Orvil™ group might have some clinical advantages over the non-Orvil™ group. We found that the Orvil™ group had a shorter operation time, less intraoperative hemorrhage and lower need for intraoperative transfusions than the non-Orvil™ group. One reason for this finding might be that the transoral placement of the anvil simplified the anastomotic procedure, and the linear-stapled transected esophageal stump improved surgical procedures and decreased the operation time. Ivor Lewis TMIE was performed independently by three senior surgeons skilled in total endoscopic esophagectomy, but different surgeons might still affect the operation time and extent of intraoperative hemorrhage.

At present, there is no consensus about the proper timing of oral feeding after MIE (13). Berkelmans et al. reported that direct oral feeding following Ivor Lewis MIE is feasible and does not increase the incidence or severity of postoperative complications or affect functional recovery (14, 15). But some surgeons postpone postoperative oral feeding time out of fear for postoperative complications such as anastomotic leakage and thoracic infection (13). Consistent with the results of a previous study (16), the patients in the Orvil™ group resumed oral feeding on postoperative day eight in this study, which was earlier than that for patients in the non-Orvil™ group. We do not believe that the significant difference in postoperative oral feeding time between the two groups is related to different anastomotic techniques. Similarly, we noted that chest tube duration was longer for the Orvil™ group than for the non-Orvil™ group in this study. This phenomenon may be a consequence of the different insights for chest tube duration in different groups. Considering that postoperative oral feeding may cause anastomotic leakage, chest tube removal was postponed in the Orvil™ group to avoid placing the chest tube again. However, the chest tube was removed earlier in the non-Orvil™ group. Surgeons in this group considered that the incidence of anastomotic leakage caused by postoperative oral feeding was relatively low after upper gastrointestinal radiography showed no abnormality.

Our data revealed that length of postoperative hospital stay was shorter for the Orvil™ group, which is consistent with the results of published study (17). We considered that multiple factors, especially severe postoperative complications, had a negative impact on the length of postoperative hospital stay. We noticed that the Orvil™ group had lower total hospitalization costs than the non-Orvil™ group in this study. Considering the different medical charge standards and anastomotic device prices of these three hospitals, there was heterogeneity in total hospitalization costs in this multicenter study. In addition, postoperative complications were the predominant factors leading to dramatically increased total hospitalization costs, where more severe complications resulted in higher hospitalization costs (18, 19). Our results showed that the total hospitalization costs of patients in the anastomotic leakage cohort were significantly higher than those in the nonanastomotic leakage cohort, which was consistent with the results reported in previous studies (18, 19). Although not all postoperative complications can be prevented, we can take appropriate preventive measures to minimize the incidence of these postoperative complications, which can not only improve the quality of life of patients but also reduce the cost of hospitalization and decrease the economic burden of patients.

Orvil™ might improve the technical safety of intrathoracic esophagogastric anastomosis during total MIE. To date, anastomotic leakage after Ivor Lewis TMIE using the Orvil™ has been reported to occur in 0-10.0% of patients (6, 16, 17, 20). In this study, the results demonstrated that the postoperative anastomotic leakage rate was 1.79% in the Orvil™ group and the Orvil™ group showed a significantly lower incidence of anastomotic leakage, which was in line with the results reported previously (7, 21). We considered that the occurrence of anastomotic leakage was multifactorial in this study and likely depended on anastomotic characteristics (anastomotic technique, anastomotic stapler quality, tension at anastomosis and internal stimulation at anastomosis), surgical factors (the surgeon’s level of surgical skills and vascular supply to the conduit), and postoperative management (psychological care, dietary guidance and rehabilitation treatment). Subsequently, we analyzed the risk factors for anastomotic leakage following Ivor Lewis TMIE, and the results showed that the anastomotic technique and pulmonary infection were independently associated with postoperative anastomotic leakage, which is inconsistent with the results previously reported by Kassis et al. (22).

Pulmonary infection was not only a common postoperative complication but also an independent risk factor for postoperative anastomotic leakage in our study. A number of previous studies have reported that preoperative respiratory comorbidities, long-term smoking and impaired preoperative pulmonary function are associated with an increased risk of postoperative pulmonary infection, which can further lead to severe malnutrition in these patients and increase the risk of anastomotic leakage (23, 24). Therefore, preoperative optimization, including exercise interventions, smoking cessation and nutritional support, should be recommended for these patients to prevent postoperative pulmonary complications and reduce the risk of postoperative anastomotic leakage.

We acknowledge that the anastomotic technique has previously been reported to be an independent factor for postoperative anastomotic leakage (4), but the optimal technique for thoracoscopic intrathoracic esophagogastrostomy remains controversial. The results of this study showed that the Orvil™ technique leads to a low anastomotic leakage rate and a high anastomotic success rate, and we consider that the Orvil™ technique should be classified as a CS side-to-side technique rather than a CS end-to-side technique, as reported in some previous studies. The process of Orvil™ anastomosis is essentially anastomosis of the side of the esophagus to the side of the gastric conduit by joining the anvil to the stapler, even though the small opening is perpendicularly close to the staple line of the esophageal stump (**Figure 2B**). Some previous studies have shown that the side-to-side technique, especially linear-stapled technique, is associated with a low rate of anastomotic leakage following esophagectomy for cancer (25–27). Therefore, the side-to-side technique might be one of the reasons for the low rate of postoperative anastomotic leakage observed in the Orvil™ group. Furthermore, we considered that the distance between the location of the small opening and the staple line of the esophageal stump might affect the quality of intrathoracic esophagogastric anastomosis (**Figure 2B**). Moreover, there may be a potential blind stump to Orvil™ anastomosis that may cause food deposition and increase the risk of infection (**Figure 2C**). However, the current data cannot confirm this hypothesis, and additional research is needed.

To date, the strength of this study lies in the large sample size, as it is the largest included in a propensity score-matched comparison between patients with esophageal cancer undergoing Orvil™ anastomosis and non-Orvil™ anastomosis. However, this study is not without limitations. First, this study was a retrospective cohort study, and the results may have been influenced by bias. Although propensity score matching can balance the observable variables, it cannot eliminate bias caused by potential unknown factors. Prospective randomized studies may be ideal methods for comparing different anastomotic techniques and further validating the application of the Orvil™ technique. Second, our data were collected in three regional public hospitals, and this multicenter clinical study may have caused potential bias due to uncontrolled factors. Third, this study focused on the effects of two different anastomotic techniques on clinical outcomes and postoperative major complications, and we did not conduct a survival analysis.

In conclusion, Orvil™ anastomosis exhibited better perioperative effects than non-Orvil™ anastomosis after propensity score-matched analysis. Remarkably, the Orvil™ technique contributed to a lower postoperative anastomotic leakage rate than the non-Orvil™ technique. With the further development of clinical research on Orvil™ anastomosis, Orvil™ technology is expected to be widely used in Ivor Lewis TMIE.

## Data Availability Statement

The original contributions presented in the study are included in the article/**supplementary Material**. Further inquiries can be directed to the corresponding authors.

## Ethics Statement

The study was approved by the Ethics Committee of the Xiangya Hospital Central South University, Tongji Hospital Affiliated to Tongji Medical College Huazhong University of Science and Technology and First Affiliated Hospital Anhui Medical University in compliance with the Declaration of Helsinki. Written informed consent was obtained from the individual(s) for the publication of any potentially identifiable images or data included in this article.

## Author Contributions

HL, CZ, and YC contributed to the conception and design of the study. CZ, HC, and YL performed these surgeries. GL collected clinical data. HL performed the statistical analysis and wrote the manuscript. All authors read and approved the final manuscript.

## Conflict of Interest

The authors declare that the research was conducted in the absence of any commercial or financial relationships that could be construed as a potential conflict of interest.

## Publisher’s Note

All claims expressed in this article are solely those of the authors and do not necessarily represent those of their affiliated organizations, or those of the publisher, the editors and the reviewers. Any product that may be evaluated in this article, or claim that may be made by its manufacturer, is not guaranteed or endorsed by the publisher.
