# Supplementary material for: Comparison of Two Circular-Stapled Techniques for Esophageal Cancer: A Propensity-Matched Analysis
Source: Front Oncol. 2021 Dec 16;11:759599. doi: 10.3389/fonc.2021.759599 (PMC8716395; doi:10.3389/fonc.2021.759599)
Supplement: Supplementary file 1 [file Table_1.docx]

**Supplemental Table 1.**  Results of Univariable Analysis of the Anastomotic Leakage and Nonanastomotic Leakage Patients

| Variables | Anastomotic leakage | Nonanastomotic leakage | *p* Value |
| --- | --- | --- | --- |
| Total, n (%) | 10 (5.3) | 177 (94.7) | - |
| Age, years | 60.5 (52.8 - 65.8) | 64.0 (56.0 - 69.0) | 0.303 |
| Sex, n (%) |  |  | 1.000 |
| Male | 9 (90.0) | 146 (82.5) |  |
| Body mass index, kg/m^2^ | 21.7 ± 2.8 | 22.5 ± 3.3 | 0.475 |
| Cigarette smoking, n (%) |  |  | 0.092 |
| Yes | 9 (90.0) | 104 (58.8) |  |
| Alcohol consumption, n (%) |  |  | 0.332 |
| Yes | 7 (70.0) | 89 (50.3) |  |
| Comorbidities, n (%) |  |  |  |
| Hypertension | 2 (20.0) | 34 (19.2) | 1.000 |
| Diabetes | 0 (0.0) | 6 (3.4) | 1.000 |
| Cerebrovascular disease | 1 (10.0) | 13 (7.3) | 0.550 |
| Peptic ulcer | 1 (10.0) | 11 (6.2) | 0.494 |
| Preoperative hemoglobin level, mg/dL | 128.0 (125.0 - 136.8) | 135.0 (123.0 - 144.0) | 0.423 |
| Preoperative albumin level, mg/dL | 38.6 ± 4.5 | 40.5 ± 5.3 | 0.278 |
| Tumor location, n (%) |  |  | 1.000 |
| Middle thoracic part | 3 (30.0) | 61 (34.5) |  |
| Lower thoracic part | 7 (70.0) | 116 (65.5) |  |
| Tumor size, cm | 3.5 (3.0 - 5.0) | 4.0 (3.0 - 5.0) | 0.705 |
| Tumor histology, n (%) |  |  | 1.000 |
| Squamous cell carcinoma | 10 (100.0) | 160 (90.4) |  |
| Adenocarcinoma | 0 (0.0) | 9 (5.1) |  |
| Other | 0 (0.0) | 8 (4.5) |  |
| Tumor stage, n (%) |  |  | 0.190 |
| 0 | 0 (0.0) | 4 (2.3) |  |
| I | 1 (10.0) | 28 (15.8) |  |
| II | 4 (40.0) | 90 (50.8) |  |
| III | 4 (40.0) | 49 (27.7) |  |
| IV | 1 (10.0) | 6 (3.4) |  |
| Length of postoperative hospital stay, days | 23.0  (19.0 - 25.3) | 12.0  (10.0 - 15.0) | <0.001 |
| Total hospitalization costs, RMB | 104788.7  (88357.9 - 125774.4) | 86068.2  (69111.4 – 102007. 5) | 0.005 |
| Anastomotic techniques |  |  | 0.016 |
| Non-Orvil^TM^ anastomosis | 8 (80.0) | 67 (37.9) |  |
| Orvil^TM^ anastomosis | 2 (20.0) | 110 (62.1) |  |
| Wound infection, n (%) | 0 (0.0) | 12 (6.8) | 1.000 |
| Anastomotic stricture, n (%) | 0 (0.0) | 6 (3.4) | 1.000 |
| Pulmonary infection, n (%) | 6 (60.0) | 5 (2.8) | <0.001 |

Values are mean ± SD, median (IQR), or n (%).
